# Auramine O, an incense smoke ingredient, promotes lung cancer malignancy

**DOI:** 10.1002/tox.22451

**Published:** 2017-07-19

**Authors:** Jia‐Chen Tung, Wei‐Chien Huang, Juan‐Cheng Yang, Guan‐Yu Chen, Chi‐Chen Fan, Yu‐Chuan Chien, Pei‐Shan Lin, Shih‐Chun Candice Lung, Wei‐Chao Chang

**Affiliations:** ^1^ Graduate Institute of Biomedical Sciences, China Medical University Taichung Taiwan; ^2^ Center for Molecular Medicine, China Medical University Hospital Taichung Taiwan; ^3^ Department of Biotechnology Asia University Taichung Taiwan; ^4^ School of Pharmacy, College of Pharmacy China Medical University Taichung Taiwan; ^5^ Chinese Medicine Research and Development Center, China Medical University Hospital Taichung Taiwan; ^6^ Department of Superintendent Office Mackay Memorial Hospital Taipei Taiwan; ^7^ Department of Medical Laboratory Science and Biotechnology Yuanpei University Hsinchu Taiwan; ^8^ Research Center for Environmental Changes, Academia Sinica Taipei Taiwan

**Keywords:** ALDH1A1, auramine O, incense, lung cancer, mass spectrometry

## Abstract

Burning incense to worship deities is a popular religious ritual in large parts of Asia, and is a popular custom affecting more than 1.5 billion adherents. Due to incomplete combustion, burning incense has been well recognized to generate airborne hazards to human health. However, the correlation between burning incense and lung cancer in epidemiological studies remains controversy. Therefore, we speculated that some unknown materials in incense smoke are involved in the initiation or progression of lung cancer. Based on this hypothesis, we identified a major compound auramine O (AuO) from the water‐soluble fraction of incense burned condensate using mass spectrometry. AuO is commonly used in incense manufacture as a colorant. Due to thermostable, AuO released from burned incenses becomes an unexpected air pollutant. AuO is classified as a Group 2B chemical by the International Agency of Research on Cancer (IARC), however, the damage of AuO to the respiratory system remains elusive. Our study revealed that AuO has no apparent effect on malignant transformation; but, it dramatically promotes lung cancer malignancy. AuO accumulates in the nucleus and induces the autophagy activity in lung tumor cells. AuO significantly enhances migration and invasive abilities and the in vitro and in vivo stemness features of lung tumor cells through activating the expression of aldehyde dehydrogenase family 1 member A1 (ALDH1A1), and ALDH1A1 knockdown attenuates AuO‐induced autophagy activity and blocks AuO‐induced lung tumor malignancy. In conclusion, we found that AuO, an ingredient of incense smoke, significantly increases the metastatic abilities and stemness characters of lung tumor cells through the activation of ALDH1A1, which is known to be associated with poor outcome and progression of lung cancer. For public health, reducing or avoiding the use of AuO in incense is recommended.

## 1 INTRODUCTION

Burning incense is a popular religious ritual and culture of worshipping activity throughout countries of Asia‐Pacific region, and this custom influences 1.5 billion adherents of major such as Hinduism, Buddhism, Taoism, and Chinese folk religions in this area.^1^ In Taiwan, there are more than 1.2 million temple goers who frequently visit temples and burn incense inside the temples.^2^ Incense stick is the most common form of incense used in Taiwan. Agarwood and sandalwood are the two major ingredients in incense, other materials such as spices, herbs, and essential oils are usually incorporated into incense components to increase the fragrance of incense smoke and make incense smolder effectively.^3^ Due to incomplete combustion, incense burning has been recognized to generate airborne hazards to human health, especially to the respiratory system. Particulate matters (PMs),^4^, ^5^, ^6^ polycyclic aromatic hydrocarbons (PAHs),^7^, ^8^, ^9^ and volatile organic compounds (VOCs)^10^, ^11^, ^12^ are hazardous materials commonly detected in incense smoke. However, the correlation between burning incense and lung tumor progression/malignancy remains controversy.^13^ Therefore, we speculated that the unidentified components in incense smoke could result in inconsistency on this issue.

Water‐soluble fraction in incense burned condensate (IBC) has long been ignored because organic matters are decomposed by combustion into small chemical compounds, most of which are organic‐soluble or volatile such as PMs, PAHs, and VOCs. Whether water‐soluble materials exist in incense smoke or they are correlated with cancer progression/malignancy is fully unknown. In this study, we utilized mass spectrometry (MS) to identify the water‐soluble materials of IBC and found auramine O (AuO) is the major compound in them. AuO (*bis*[4‐(dimethylamino)phenyl] methaniminium chloride), a yellow dye, is commonly used in incense manufacture as a colorant. AuO has a high melting point of 267°C and thermostability, which results in the existence of AuO in incense smoke as an unexpected air pollutant.

Oral administration of high‐dose AuO has been shown to induce liver tumors in both mice and rats^14^ and cause genotoxicity in primary rat and human hepatocytes,^15^ and the International Agency of Research on Cancer (IARC) classified AuO into Group 2B as a possible carcinogen to humans.^16^ To date, the exposure and damage of AuO to the respiratory system has never been recognized. Therefore, we investigated the carcinogenic potential of AuO for the initiation and progression of lung tumor. In this study, we found that AuO undergoes nuclear translocation and induces autophagy activity in lung tumor cells. AuO has no apparent effect on inducing lung tumor initiation. However, AuO significantly increases cell migration and invasive abilities through activating the stemness of tumor cells, which finally leads to tumor malignancy.

## 2 MATERIAL AND METHODS

### 2.1 Chemicals, antibodies, and plasmids

Acridine orange (#A9231) and auramine O (#861030) were purchased from Sigma‐Aldrich. The antibodies used in this study include ALDH1A1 (#12035; Cell Signaling), CD133 (#PAB12663; Abnova), CD44 (#LS‐C44932; LSBio), LC3B (#ab51520; abcam), and β‐actin (#3700; Cell Signaling). The shRNAs against ALDH1A1 gene were purchased from the National RNAi Core Facility (Academia Sinica, Taipei, Taiwan). The target sequences of shALDH1A1#1 and shALDH1A1#2 are: CACCGATTTGAAGATTCAATA and GCTGATTTAATCGAAAGAGAT, respectively.

### 2.2 Incense and incense smoke collection

Incense sticks including Chen‐Shiang (CS; agarwood) and Tan‐Shiang (TS; sandalwood) were purchased from vendors surrounding Dajia Jenn Lann Temple (Taichung, Taiwan) and local incense manufacture (Taichung, Taiwan). Incense sticks (three CS and three TS) collected from vendors were used for the estimation of AuO content, whereas those (two CS and two TS) collected from local incense manufacture were used as reference because their AuO content was no detection. The sampling device and sampling method have been described in an earlier publication.^17^ Personal environmental monitor (SKC 761–203) with a size cut of 2.5 μm mounted with 37 mm Teflon filter (SKC 225–1709) was used for smoke particle sampling. After 5 min collection, the filters were cut into small pieces and extracted by 20 mL ddH_2_O. The extracted solution was lyophilized and resolved with assigned volume of ddH_2_O.

### 2.3 Mass spectrometry

Materials extracted from IBC were assayed by Apex Qe FT‐ICR MS equipped with a 9.4 T actively shielded magnet (Bruker Daltonics). The analysis was set in positive ion mode using an Apollo II electrospray source. The MS scan was set *m*/*z* 100 to 1000 with a mass resolution of 66,000 at *m/z* 400. The spectra were processed using Data Analysis 4.0 (Bruker Daltonics). For comparative proteomic analysis, the trypsinized peptides were identified using the linear ion trap‐Fourier transform ion cyclotron resonance mass spectrometer (LTQ‐FTICR MS, Thermo Fisher). The survey scan of MS analysis (*m/z* 320–2000) was performed on LTQ‐FTICR MS with a mass resolution of 100,000 at *m/z* 400. Top 10 most abundant multiply charged ions were sequentially isolated for MS/MS by LTQ. The MaxQuant^18^ and MaxLFQ^19^ software were used for protein identification and label‐free quantification. The significance threshold for protein identification was set to *P* < .01.

### 2.4 Molecular modeling

The molecular modeling was performed to predict the binding affinity of AuO to DNA using Autodock software, and the similar structures of AuO were analyzed using Autodock 4.2 software with Lamarckian Genetic Algorithm.^20^ The crystal structure of DNA was obtained from the protein data bank (PDB ID: 1Z3F) (http://www.rcsb.org/pdb/home/home.do).^21^ The substrates including ligands, water, and small molecules were removed from co‐crystallized DNA. Polar hydrogens and Kallman united atom charges were added to the DNA for docking calculation using Autodock Tool 1.5.6 interfaces (ADT).^22^, ^23^ The optimization of AuO and its similar compounds were performed by using MMFF94 force field by ChemBio3D software (version 11.0; Cambridge Soft Corp.). Hydrogens and Gasteiger charges were added to the AuO for docking.^22^ The Grid box calculated by AutoGrid program was centered at the binding site of co‐crystal ligand of DNA with dimensions 68 × 64 × 44 Å grid points at spacing of 0.375Å. All docking parameters were set to default except for the following parameter: maximum number of energy evaluation increase to 25,000,000 per run. The docking results were analyzed by using cluster analysis and were shown by ADT. The models of docking results were shown by Discovery Studio Visualizer 4.5 (Accelrys).

### 2.5 Isothermal titration calorimetry

The energetic process of AuO‐dsDNA association was measured by the isothermal titration calorimetry (ITC, TA Instrument). The titration of dsDNA (five GC pairs of DNA) with AuO was performed by adding AuO from a rotating syringe in 20 injections with an interval of 310 s. All solutions were appropriately degassed to avoid the interference of bubble formation during the process of titration. The measurement in phosphate buffer was used as the background control to correct for the heat of dilution and deconvoluted on NanoAnalyze software (version 2.4.1) according to a single site binding model.

### 2.6 Cell culture

The human nontumorigenic bronchial epithelial cell line BEAS‐2B was cultured in DMEM/F‐12 medium [with 1 μM hydrocortisone, 5 μg/mL insulin, 10 μM HEPES, 10% fetal bovine serum (Gibco), and 1% penicillin/streptomycin (Gibco)]. The human lung adenocarcinoma cell line CL1–0 was gifts from Dr. Pan‐Chyr Yang (National Taiwan University, Taipei, Taiwan). Both CL1–0 and A549 cells were maintained in RPMI 1640 media (Invitrogen) supplemented with 10% fetal bovine serum and 1% antibiotics (Gibco). All cells were grown in a humidified atmosphere of 5% CO_2_ and 95% air at 37°C.

### 2.7 Protein extraction and tryptic peptide preparation

Total proteins were extracted using RIPA lysis and extraction buffer (Thermo Fisher) and protein quantification was determined using the Bio‐Rad Protein Assay by the measurement of absorbance at 595 nm. Total 20 μg of protein was separated using 10% SDS‐PAGE and divided into 10 gel fractions, which were then cut into small gel pieces (<1 mm^3^) individually. The in‐gel digestion procedure includes the following steps in order: (1) destaining using 50% acetonitrile (ACN) and 25 mM ammonium bicarbonate (ABC); (2) reduction using 10 mM dithiothreitol at 58°C for 45 min; (3) alkylation using 55 mM iodoacetamide at RT for 45 min in the dark; (4) enzyme digestion using 4 ng/μL sequencing grade trypsin in 25 mM ABC solution at 37°C for 16 h; (5) extraction of tryptic peptides using a 60% ACN/1% trifluoroacetic acid solution. After drying to remove the solvent, the tryptic peptides were dissolved and used for MS.

### 2.8 Western blot analysis

After SDS‐PAGE separation, proteins were transferred onto a PVDF membrane using electroblot at 400V at 4°C for 3 h in 25 mmol/L Tris‐HCl, 197 mmol/L glycine, and 13.3% (v/v) methanol. Membranes were blocked with 5% (w/v) skim milk in TBST for 1 h, and then incubated with primary antibodies by a gentle shaking at room temperature for overnight. After gently agitating in three TBST washes and one TBS wash for 15 min each, horseradish peroxidase (HRP)‐conjugated secondary antibody was added to further incubate at RT for 1 h. Immunoreactive signals were revealed using an enhanced chemiluminescence substrate kit (NEN Life Science), which includes luminescent substance luminol that is oxidized to luminesce by HRP using H_2_O_2_ as an oxidizing agent and enhancers that increases the light intensity, and the signals were recorded by developing photographic film under optimum exposure.

### 2.9 Wound healing and matrigel invasion assay

The wound healing assay was used to study the migration ability of tumor cell in vitro. Tumor cells (70 µL; concentration: 3 × 10^5^ cells/mL) were added to Culture‐Insert well (ibidi) and cultured for 24 h. A “wound gap” was created by removal of Culture‐Insert, and the “healing” of this gap by cell migration was recorded per 4 h until 24 h. The migration area of tumor cells was measured using Image J software. The matrigel invasion assay was used to determine the invasion ability of tumor cell in vitro. Tumor cells (2.5 × 10^5^ cells in 200 µL) were suspended in DMEM medium and added to the upper half of a PET membrane transwell insert chamber (BD Biosciences), which was coated with Matrigel (1 mg/mL; BD Biosciences) on a 24‐well plate. DMEM medium supplemented with 10% FBS was added as a chemoattractant to the lower half. After incubation at 37°C for 24 h, tumor cells that passed through the insert were fixed with 3% formalin (Sigma‐Aldrich) and stained with 0.2% crystal violet (Sigma‐Aldrich).

### 2.10 Spheroid formation assay

Spheroid formation assay was performed in 6 cm culture dish coated with 1% agarose. Tumor cells suspended in serum free medium were seeded at a density 5000 cells/dish and incubated in a humidified atmosphere of 5% CO_2_ and 95% air at 37°C for 7 days. The numbers of spheroid were counted manually at 7th day under microscope.

### 2.11 Mouse model of subcutaneous inoculation

The animal procedure (2016–047) was approved by the Institutional Animal Care and Use Committee (IACUC) at CMUH. Lung cancer cell A549 was pretreated with 1 μM AuO for 7 days. Both AuO‐treated and control A549 cells (1 × 10^6^) were mixed with matrigel and injected into 5‐week‐old male BALB/c nude mice (purchased from BioLASCO, Taiwan). Left flank was injected with control, whereas right flank was injected with AuO‐treated A549 cell. Tumor size was measured by caliper weekly for 6 weeks once tumors became visible. Tumor volume was calculated with the formula: (length × width^2^)/2. After animal sacrifice, tissue samples were fixed in formalin and embedded with paraffin, and then analyzed using immunohistochemical (IHC) staining. The IHC procedure followed our previously described method.^24^ Briefly, rabbit anti‐human ALDH1A1 antibody (#12035; Cell Signaling) was used to perform IHC staining by horseradish peroxidase‐conjugated avidin‐biotin complex from the Vectastain Elite ABC Kit (Vector Laboratories) and AEC chromogen (Vector Laboratories).

### 2.12 Statistical analysis

Data are expressed as means ± SD. The significance of difference was examined by Student's *t*‐test (two‐tailed). *P* < .05 was considered to be significant.

## 3 RESULTS

### 3.1 AuO is the major water‐soluble component of IBC

**Figure 1:**
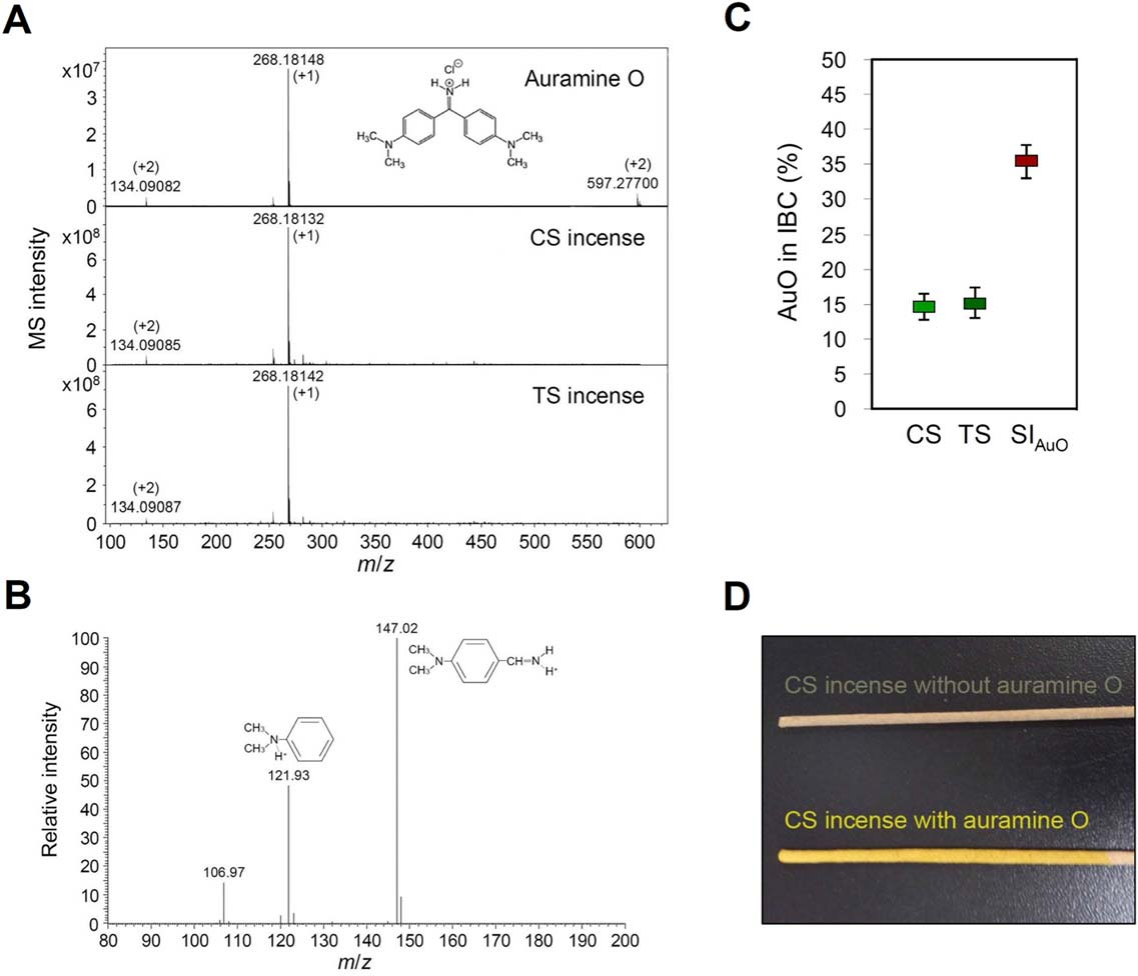
AuO is the major water‐soluble component of IBC. A, The compositions of commercial standard AuO and water‐soluble IBC compounds extracted form CS and TS incenses were analyzed by an Apex Qe FT‐ICR MS. B, Tandem mass profile of parental ion *m/z* 268.18 was generated by a Velos Pro MS. C, The weight percentage of AuO in commercial and AuO‐soaked incenses was determined by MS signal intensity derived quantitative calibration curve. D, The different appearances between incenses with and without AuO [Color figure can be viewed at wileyonlinelibrary.com]

The Personal Environmental Monitor (SKC Inc., PA) was used to collect the soot materials emitted from commercial Chen‐Shiang (CS, agarwood) and Tan‐Shiang (TS, sandalwood) incenses.^17^ The water‐soluble components were extracted from Teflon membranes (PM_10_ was collected) and analyzed using an Apex‐Qe Fourier transform ion cyclotron resonance mass spectrometer (Bruker Daltonics). A major compound with *m*/*z* 268.18 is detected in both incense samples (Figure 1A), and it has two daughter fragments with *m*/*z* 122 and *m*/*z* 147 in the tandem mass spectrum (Figure 1B). The chemical formula of target compound was deduced from the precise molecular weight of parent ion and the tandem mass signals of daughter ions, the search result showed that the potential candidate is AuO. Using commercial standard AuO, we confirmed that it exhibits the consistent mass signal (Figure 1A, upper panel) and tandem mass signal with the compound extracted form IBC.

To quantify the content of AuO in IBC and incense, we used AuO standard compound to construct a calibration curve using a Velos Pro MS (Thermo Fisher) coupled with a nano‐HPLC system. The linear relationship for AuO quantity and peak‐area intensity is calculated as *y* = 8.95*x* + 3044 and an *R* ^2^ of 0.9965, with AuO quantity ≧10 femtomole. Based on this linear equation, the content of AuO in CS and TS incenses is calculated as 5.25 mg/g and 4.85 mg/g (average concentration), respectively. The weight percentage of AuO in IBC of CS and TS incenses is 14.4% and 14.9%, respectively (Figure 1C). In addition, we soaked AuO‐free incenses with the saturated AuO solution (Figure 1D), which results in an up to 35.4% of weight percentage of AuO in IBC (Figure 1C).

According to our previous study,^25^ the average exposure concentration of PM_10_ in temples was around 600 μg/m^3^, approximately equal to 88 μg/m^3^ of AuO (assuming AuO accounts for 14.7% of PM_10_). Consequently, we estimate that a temple worker (8 h working time per day) inhales 338 μg (1.27 μmol) of AuO into the lung, and the maximum accumulation concentration in blood is 289 nM in 1 day (Supporting Information Table 1).

### 3.2 AuO accumulates in the nucleus and binds DNA of lung tumor cells

**Figure 2:**
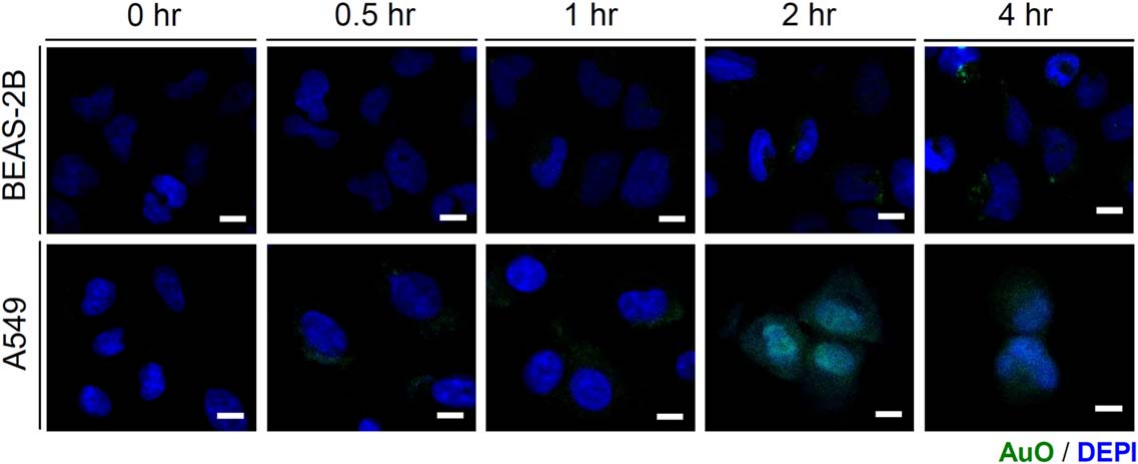
AuO accumulates in the nucleus of A549 cells. Human bronchial epithelial cell BEAS‐2B and lung tumor cell A549 were treated with 10 μM AuO for different times. The accumulation of AuO in cells was observed by confocal microscopy. Scale bars, 10 μm [Color figure can be viewed at wileyonlinelibrary.com]

**Figure 3:**
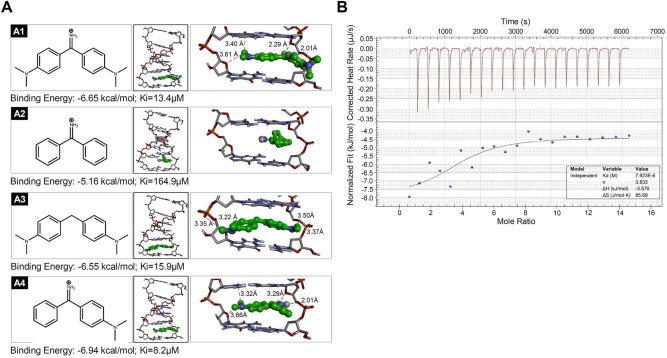
AuO binds the genomic DNA. A, The binding model of four simulated AuO derivatives in DNA. Compound A1–A4 (green ball‐stacked form) gets the docked binding energy values −6.65, −5.16, −6.55, and −6.94 kcal/mol, and Ki values 13.4, 164.9, 15.9, and 8.2 μM, respectively. The binding affinity of compound to DNA is dependent on the hydrogen bond (green dash line) interacted with DNA double helix. Compound A1 forms four hydrogen bonds with bond length 2.01 Å, 2.29 Å, 3.40 Å, and 3.61 Å. Compound A2 does not form any hydrogen bond. Compound A3 forms four hydrogen bonds with bond length 3.22 Å, 3.35 Å, 3.37 Å, and 3.50 Å. Compound A4 forms four hydrogen bonds with bond length 2.01 Å, 3.29 Å, 3.32 Å, and 3.66 Å. B, Isothermal titration calorimetry (ITC) was used to determine the binding of AuO to five pairs of dsDNA (GCs) at pH 7.0. The upper figure shows raw data curve and the down figure shows the fitted integrated data curve [Color figure can be viewed at wileyonlinelibrary.com]

To understand the mode of action of AuO in the lung, we first observed the cellular location of AuO based on its fluorescent feature using confocal microscopy. After AuO treatment, AuO enters tumor cell and accumulates in the nucleus of A549 within 2 h (Figure 2), suggesting the DNA binding potential of AuO. However, the nuclear translocation is not observed in bronchial epithelial cell BEAS‐2B up to 4 h, implying normal lung cells might be more resistant to AuO (Figure 2). Further, we conducted the molecular modeling to understand the motif of AuO is essential for DNA interaction. As shown in Figure 3A, the methylamino group is important for DNA binding ability of AuO (A4 compound Ki: 8.2 μM vs. A2 compound 164.9 μM). However, the dimethylamino group is not essential for enhancing the binding affinity (A4 compound 8.2 μM vs. A1 compound 13.4 μM). Interestingly, the iminum group of AuO does not affect the interaction of AuO‐dsDNA (A1 compound 13.4 μM vs. A3 compound 15.9 μM). To analyze the binding affinity of AuO to DNA, isothermal titration calorimetry (ITC) was used for assessing the change in Gibbs free energy during the interaction of AuO and double‐strand DNA through calculating the thermodynamic parameters of interactions in solution. The dissociation constant for AuO and double strand DNA is 7.9 μM (Figure 3B). In summary, these results point out the nuclear translocation capability and DNA binding ability of AuO, especially in lung tumor cells.

### 3.3 AuO induces lung tumor cell autophagy

**Figure 4:**
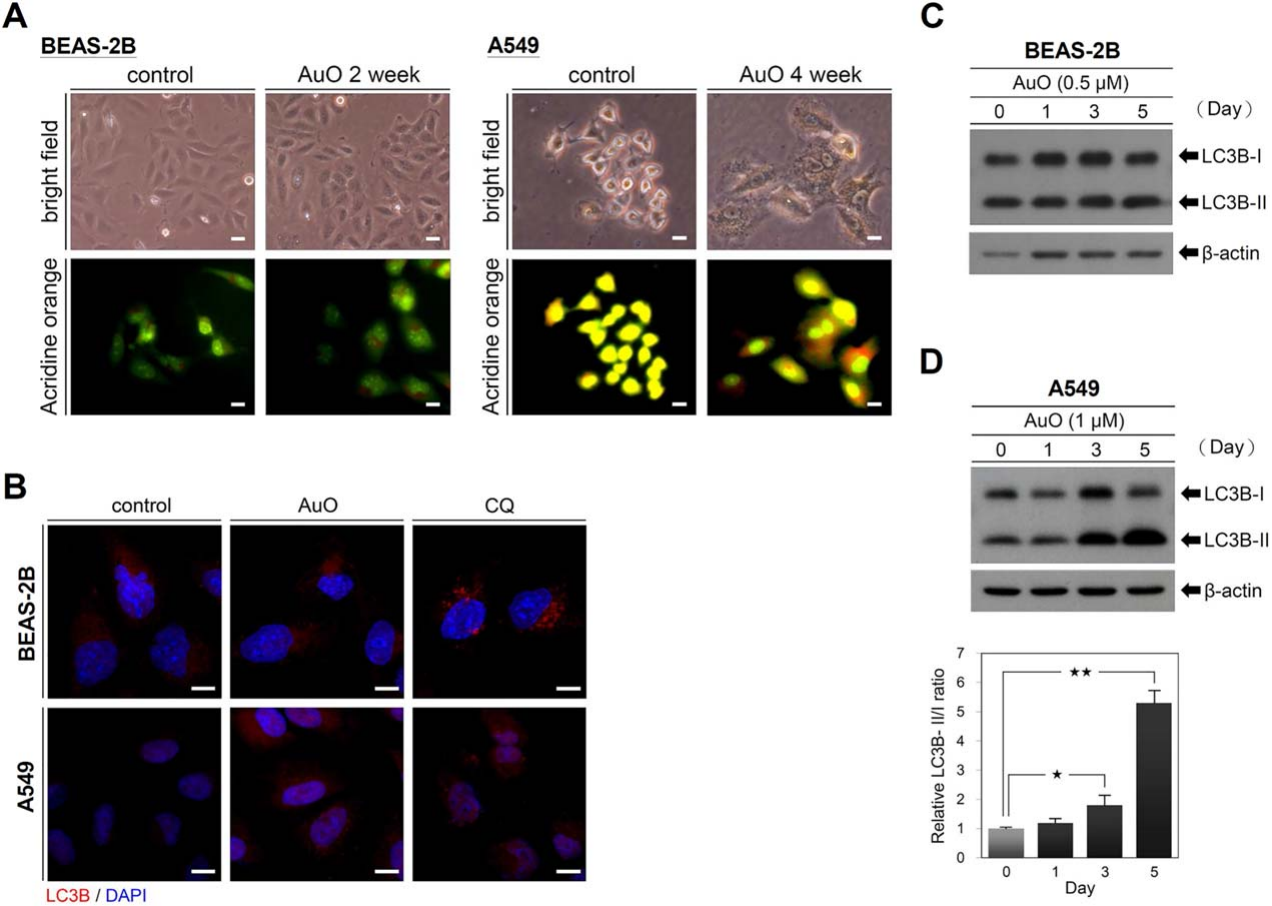
AuO induces autophagy in A549 cells. A, The morphologies of BEAS‐2B and A549 without and with AuO treatment (0.5 μM AuO for BEAS‐2B and 1 μM AuO for A549) were observed by a bright‐field microscopy. The staining of acridine orange was observed by a fluorescent microscopy. B, Confocal images of BEAS‐2B and A549 with antibody to LC3B. Both cells were treated with AuO for 3 days or with 150 μM chloroquine for 2 h. C, Western blot analysis of LC3B in BEAS‐2B. D, Western blot analysis of LC3B in A549. The treated times of AuO as indicated. Relative LC3B‐II/I ratio was analyzed by software image J. β‐actin, loading control [Color figure can be viewed at wileyonlinelibrary.com]

In comparison to normal culture condition, BEAS‐2B cells do not have remarkably morphological change under AuO‐containing culture condition (Figure 4A). However, AuO treatment leads to the granular structures at the surface of A549 cells (Figure 4A). We speculated that the granular structures might be AuO‐induced autophagosomes. To verify this hypothesis, acridine orange was used to stain the autophagosomal vacuoles based on their acidic feature. The AuO‐treated A549 cells exhibit stronger orange to red fluorescent signals compared with untreated control cells, but BEAS‐2B cells do not show significant fluorescent change (Figure 4A). The microtubule‐associated proteins 1 light chain 3B (LC3B) is an essential autophagosomal protein and LC3B puncta represent a convenient marker of autophagy.^26^ To confirm AuO induces autophagy activity, we compared the protein expression of LC3B in both BEAS‐2B and A549 cells with or without AuO treatment. Confocal images showed that an increase of LC3 puncta is observed in AuO‐treated A549 cells in comparison to the control cells (Figure 4B). Chloroquine (CQ) treatment is used as positive control because CQ inhibits both fusion of autophagosome with lysosome and lysosomal protein degradation, which leads to LC3B accumulation.^27^ In addition, significantly higher levels of LC3B II/I ratio (a definitive autophagy biomarker) were observed in A549 cells within 5‐day AuO treatment (Figure 4D). However, AuO does not have an obvious effect on enhancing autophagy activity in bronchial epithelial cell BEAS‐2B (Figure 4B,C). These results are consistent with the confocal observations. Collectively, our data demonstrate that AuO exposure increases autophagy activity of lung cancer cell.

### 3.4 AuO has no apparent effect on lung cancer initiation

**Figure 5:**
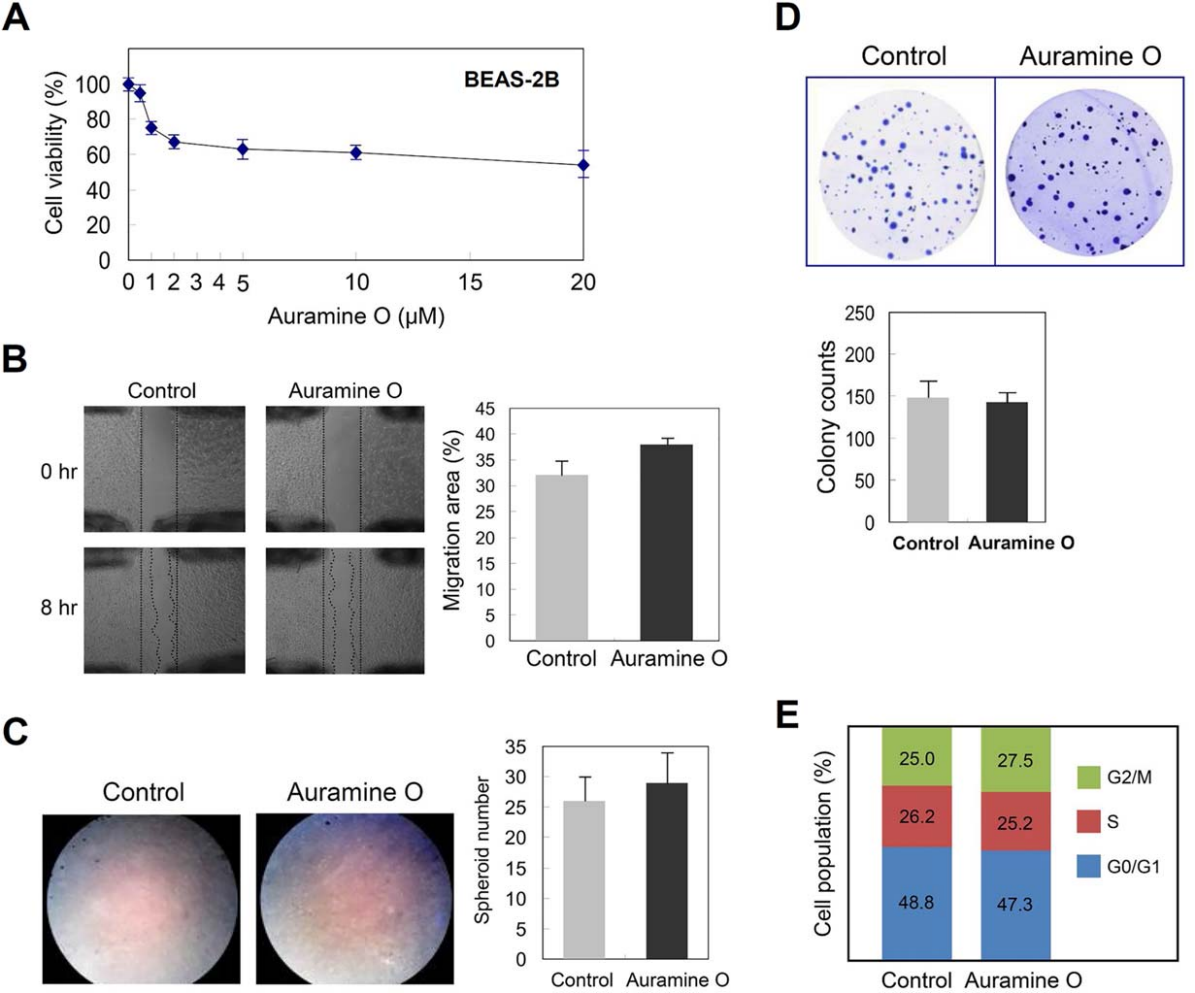
AuO has no apparent effect on promoting neoplastic transformation of BEAS‐2B. A, BEAS‐2B cells were treated with various doses of AuO for 1 day and the cell viability was determined by MTT assay. Before performing the following analyses B to E, BEAS‐2B was pretreated with 0.5 μM AuO for 7 days. B, Wound healing assay to determine the migration ability of cells in vitro. C, Transwell assay to determine the invasion ability of cells in vitro. D, Soft agar assay. BEAS‐2B cells (2 × 10^4^/well) were cultured in 0.3% agarose gel (based agarose gel 0.6%), and cell colonies were stained with crystal violet. E, The cell cycle was analyzed by flow cytometry. The illustration showed cell population in distinct stages of cell cycle. Experiments were performed in triplicate (mean ± SD) [Color figure can be viewed at wileyonlinelibrary.com]

Since AuO is known as a possible carcinogen to humans, we wonder whether AuO promotes lung cancer initiation. To determine the effect of AuO on lung cell transformation, BEAS‐2B cells were treated with various doses of AuO, and the result showed that AuO at 0.5 µM (the maximal concentration) does not dramatically affect cell viability (Figure 5A). After 7‐day AuO treatment, BEAS‐2B cells do not significantly change cell migration and invasive abilities (Figure 5B,C), nor the ability of anchorage‐independent growth in soft agar (Figure 5D). The cell cycle of BEAS‐2B is also unchanged under AuO treatment (Figure 5E). Combined with the previous observations, all our findings suggest that normal lung cells exhibit insensitive to AuO stimulation and AuO could be an ineffective carcinogen to directly induce normal lung cell transformation, at least at the current experimental condition.

### 3.5 AuO promotes lung cancer malignancy

**Figure 6:**
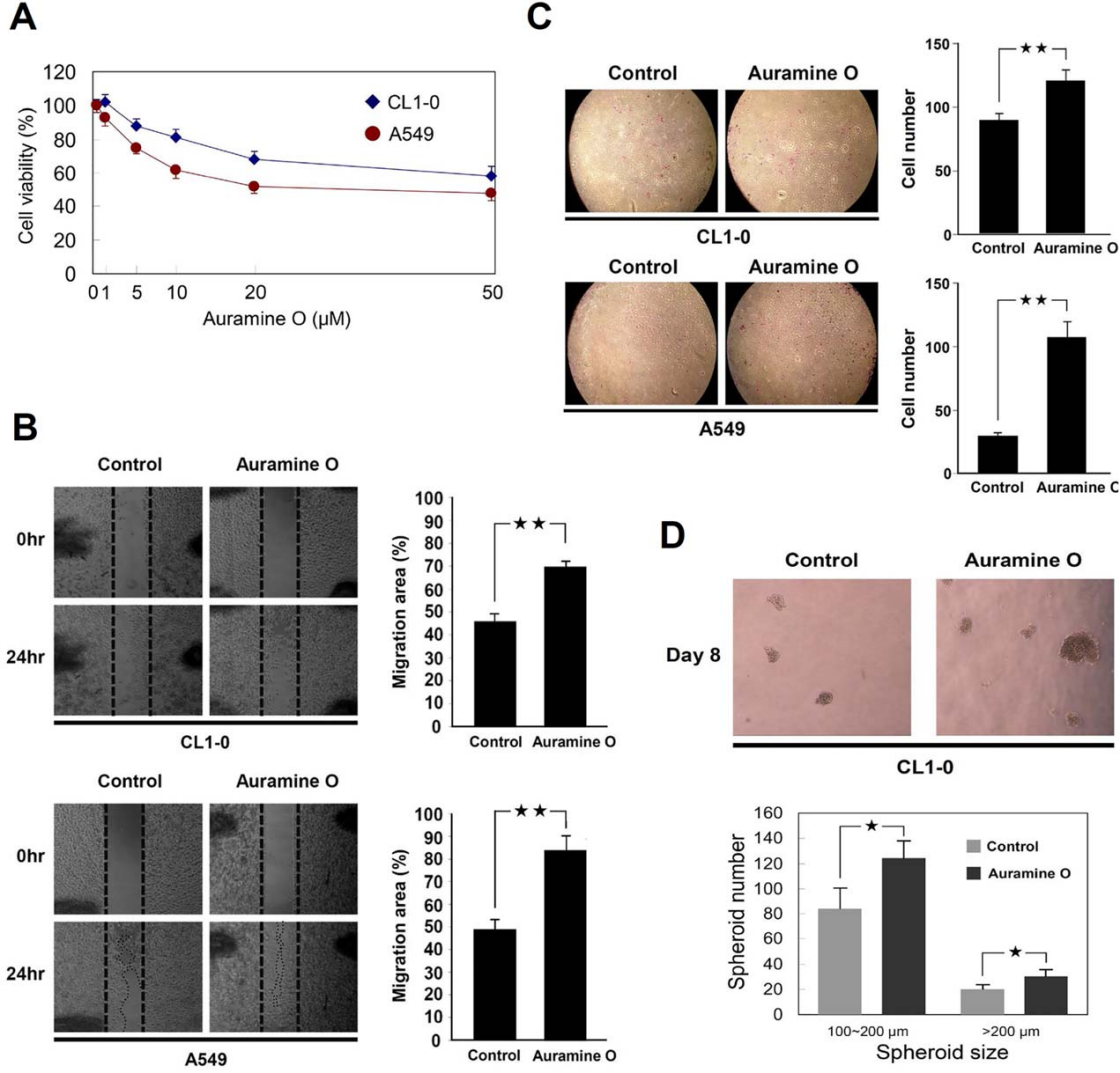
AuO promotes lung cancer malignancy. A, A549 and CL1–0 cells were treated with various doses of AuO for 1 day and the cell viability was determined by MTT assay. After pretreated with 1 μM AuO for 7 days, AuO‐treated and the control A549 and CL1–0 cells were used to perform the following analyses. B, Wound healing assay. C, Transwell assay. D, Spheroid formation assay. Experiments were performed in triplicate (mean ± SD). *P < .05; **P < .01 [Color figure can be viewed at wileyonlinelibrary.com]

Next, we determined whether AuO promotes lung cancer malignancy. Two human lung cancer cell lines A549 and CL1–0 were used to examine their metastatic abilities after AuO treatment. Various doses of AuO were tested and 1 µM AuO was used for the following treatment because this dose shows a limited inhibition to cancer viability (Figure 6A). After 7 culture days, both cancer cells increase their migration and invasive abilities (Figure 6B,C). Moreover, AuO enhances the spheroid formation of lung cancer cells in number and size (Figure 6D), suggesting that AuO could increase the stemness of lung tumor cells. These data indicate that AuO significantly promotes lung cancer malignancy.

### 3.6 AuO increases the metastatic abilities of lung tumor cells through activating ALDH1A1 expression

**Figure 7:**
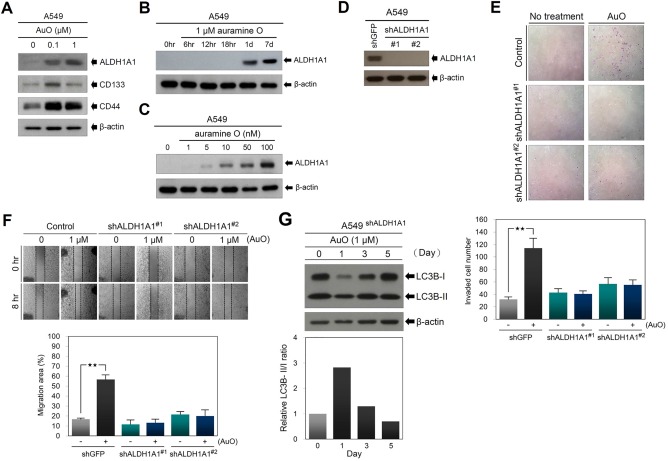
AuO enhances the metastatic abilities of A549 cells through increasing ALDH1A1 expression. A, A549 cells were treated with indicated dose of AuO and the expression of ALDH1A1, CD133, and CD44 was determined by western blot. B, Western blot assay of ALDH1A1 in A549 cells treated with 1 μM AuO at different times. C, Western blot assay of ALDH1A1 in A549 cells treated with various doses of AuO for 1 day. D, Western blot assay of ALDH1A1 in A549 cells was used to validate the shRNA knockdown efficiency. E, Transwell assay was used to determine the effect of ALDH1A1 knockdown on cell invasion of A549. F, Wound healing assay was used to determine the effect of ALDH1A1 knockdown on cell migration of A549. G, Western blot analysis of LC3B in AuO‐treated A549 cells with ALDH1A1 knockdown. β‐actin, loading control. **P < .01 [Color figure can be viewed at wileyonlinelibrary.com]

To investigate the molecular effect of AuO on lung cancer malignancy, a comparative proteomic analysis was performed to determine the protein changes between AuO‐treated A549 cells and their control cells. Total 2712 proteins are identified, and the overexpressed proteins in AuO‐treated cells with a fold increase of more than five are listed in Table 1. Of these, we noticed that aldehyde dehydrogenase family 1 member A1 (ALDH1A1), which has been characterized as a potential cancer stem cell (CSC) marker of lung cancer and its expression is associated with poor outcome and progression of lung cancer.^28^, ^29^ Western blot validation showed that AuO induces ALDH1A1 expression in a dose‐dependent manner (Figure 7A). Although other CSC‐related markers such as CD133 and CD44 were not included in the list, they also showed an increase of protein levels under AuO treatment in western blot assay (Figure 7A). To evaluate the efficiency of AuO in ALDH1A1 induction, the shortest time and minimum dose of AuO treatment were determined in A549 cells. As presented in Figure 7B,C, AuO is able to induce ALDH1A1 at the minimum dose of 5 nM within 1 day, which is an achievable dose for people under daily exposure based on our previous estimation in temple workers.

**Table 1:** The overexpressed proteins in AuO‐treated A549 cells in comparison with the control cells

Uniprot	Protein name	Gene name	Unique no.	Coverage (%)	MW (kDa)	PEP^a^
P00352	Aldehyde dehydrogenase family 1 member A1	ALDH1A1	25	62.9	54.86	<1.0E‐307
B9EGG0	Anoctamin‐6	ANO6	3	4.4	108.43	6.19E‐06
P10398	Serine/threonine‐protein kinase A‐Raf	ARAF	1	4.1	67.93	3.78E‐03
O75787	Renin receptor	ATP6AP2	5	19.7	39.01	1.93E‐12
O60826	Coiled‐coil domain‐containing protein 22	CCDC22	4	10.4	70.76	6.81E‐56
Q9UFE4	Coiled‐coil domain‐containing protein 39	CCDC39	2	2.7	109.90	9.11E‐04
Q8WVB6	Chromosome transmission fidelity protein 18 homolog	CHTF18	3	3.6	129.40	1.99E‐13
Q8N668	COMM domain‐containing protein 1	COMMD1	3	27.4	21.18	3.23E‐11
Q9H9Q2	COP9 signalosome complex subunit 7b	COPS7B	3	19.7	29.62	5.90E‐13
Q13618	Cullin‐3	CUL3	4	9.4	88.93	1.10E‐40
P63172	Dynein light chain Tctex‐type 1	DYNLT1	2	30.1	12.45	2.60E‐11
O43432	Eukaryotic translation initiation factor 4 gamma 3	EIF4G3	5	8.5	180.50	6.38E‐17
Q9NYY8	FAST kinase domain‐containing protein 2	FASTKD2	3	7.0	81.46	6.04E‐65
Q86VS8	Protein Hook homolog 3	HOOK3	3	5.0	83.13	6.13E‐09
P56937	3‐keto‐steroid reductase	HSD17B7	3	12.3	38.21	3.35E‐11
P42858	Huntingtin	HTT	7	3.6	347.86	2.18E‐174
Q86VI3	Ras GTPase‐activating‐like protein IQGAP3	IQGAP3	5	5.6	184.70	2.16E‐13
Q12768	WASH complex subunit strumpellin	KIAA0196	6	7.4	134.28	4.20E‐14
Q92845	Kinesin‐associated protein 3	KIFAP3	2	3.9	91.20	1.52E‐03
P45985	Dual specificity mitogen‐activated protein kinase kinase 4	MAP2K4	3	17.0	44.29	1.70E‐16
O95983	Methyl‐CpG‐binding domain protein 3	MBD3	3	14.8	32.84	8.06E‐11
O15091	Mitochondrial ribonuclease P protein 3	MRPP3	4	8.9	67.32	1.19E‐04
Q8NC60	Nitric oxide‐associated protein 1	NOA1	3	7.3	78.46	9.84E‐121
P37198	Nuclear pore glycoprotein p62	NUP62	3	10.0	53.25	5.08E‐14
P19174	1‐Phosphatidylinositol 4,5‐bisphosphate phosphodiesterase gamma‐1	PLCG1	4	4.0	148.66	1.18E‐07
Q8TF05	Serine/threonine‐protein phosphatase 4 regulatory subunit 1	PPP4R1	4	6.7	107.00	8.23E‐24
P49643	DNA primase large subunit	PRIM2	3	7.3	58.81	1.94E‐04
P34896	Serine hydroxymethyltransferase, cytosolic	SHMT1	3	12.4	53.08	6.27E‐10
Q05519	Serine/arginine‐rich splicing factor 11	SRSF11	3	8.1	53.54	1.32E‐03
Q8TC07	TBC1 domain family member 15	TBC1D15	5	8.5	79.49	1.78E‐08
Q00059	Transcription factor A, mitochondrial	TFAM	3	9.8	29.10	3.00E‐03
Q9UPU5	Ubiquitin carboxyl‐terminal hydrolase 24	USP24	14	7.7	294.36	9.70E‐25

a Posterior error probability (PEP) was obtained from statistical analysis of total peptide identification for a protein in one sample. The value essentially operates as a statistical value, and low PEP indicates high statistical significance.

To address whether AuO increases lung cancer metastatic abilities through ALDH1A1 activation, small hairpin RNAs (shRNAs) were used to knock down ALDH1A1 expression in A549 cells. Western blot confirmed that two shRNAs inhibit AuO‐induced ALDH1A1 expression (Figure 7D) and significantly abolish AuO‐induced migration and invasive abilities in A549 cells (Figure 7E,F). Moreover, because autophagy has been shown to be involved in modulating tumor cell motility and invasion,^30^ we checked the effect of ALDH1A1 knockdown on AuO‐induced autophagy activity. Compared with the data in Figure 4D, ALDH1A1 knockdown attenuates AuO‐induced autophagy activity (Figure 7G). These results suggest that ALDH1A1 plays an important role in mediating AuO‐induced lung cancer metastasis.

### 3.7 AuO enhances the stemness of lung tumor cells in vitro and in vivo through the induction of ALDH1A1 expression

**Figure 8:**
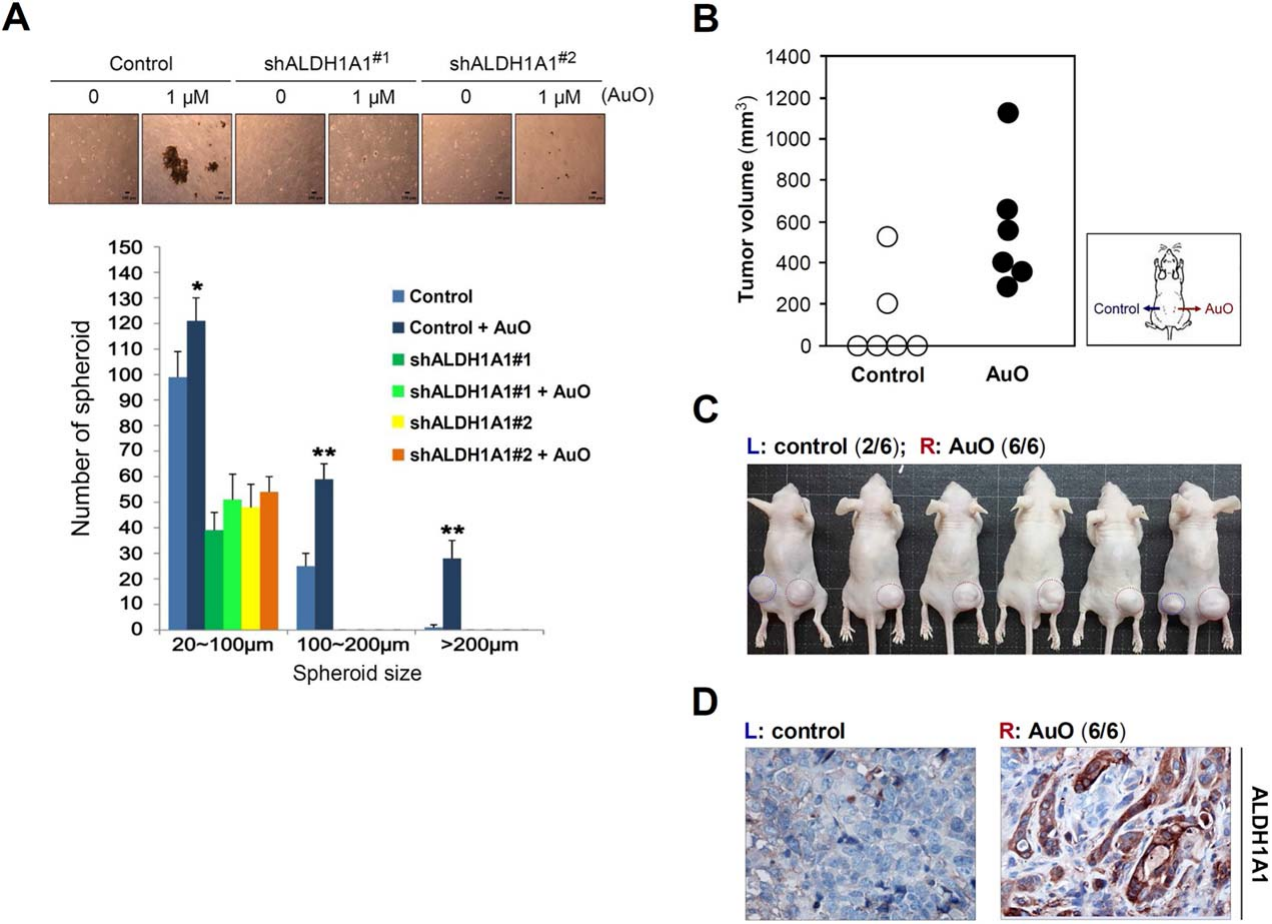
AuO enhances the stemness feature of A549 cells. A, spheroid formation assay was used to determine the effect of ALDH1A1 knockdown on in vitro stemness feature of A549 cells. Various sizes of spheroids in A549 cells with and without AuO treatment were counted at day 8. *P < .05; **P < .01. B, AuO‐treated and the control A549 cells (1 × 10^6^) were subcutaneously injected into both flanks of 5‐week‐old male BALB/c nude mice (*N* = 6) as indicated. Tumor volume was calculated with the formula: (length × width^2^)/2. The tumor volumes show the measurement after 6‐week injection. C, The formed tumors in experimental mice after 6‐week injection. D, Representative IHC images of ALDH1A1 expression in AuO‐treated and the control tumor tissues. [Color figure can be viewed at wileyonlinelibrary.com]

To determine whether ALDH1A1 is essential for AuO‐induced stemness features, we examined the effect of ALDH1A1 knockdown on in vitro stemness character by spheroid formation assay. The result showed that the spheroid number and size of A549 cells are significantly attenuated by ALDH1A1 knockdown (Figure 8A). To evaluate whether AuO increases in vivo tumorigenesis, we subcutaneously injected control (shGFP) and AuO‐pretreated A549 cells into both flanks of immunodeficient mice and monitored tumor growth. The data showed that AuO treatment promotes tumorigenesis of A549 cells and increases the speed of tumor growth (Figure 8B,C). Additionally, ALDH1A1 has higher expression levels in tissues of AuO‐treated mice in comparison with the control in the IHC analysis (Figure 8D). Taken together, these results demonstrate that AuO enhances the stemness characters of lung tumor cell by increasing ALDH1A1 expression.

## 4 DISCUSSION

AuO is a multifunctional dye used in the coloring of paper, textile, leather, incense as well as acid‐fast bacteria such as *Mycobacterium tuberculosis*.^31^ Due to low price, high intensive color, and excellent stability, AuO has been found to extensively add in foodstuff and pharmaceuticals as an illegal additive. The abuse of AuO is regarded as an important public health issue because of its carcinogenic potential.^15^ Therefore, AuO is forbidden in food matrices by many international food regulation acts, including European Union,^32^ China,^33^ Japan,^34^ and the United States.^35^ Meanwhile, many sensitive and rapid methods are developed for the detection and removal of AuO to ensure a safe living environment.^36^, ^37^, ^38^ However, whether AuO damages the respiratory system remains fully unknown. In this study, we identified AuO from water‐soluble fraction of IBC as unexpected air pollution. AuO is able to induce the stemness of lung tumor cells at the minimum concentration of 5 nM within 1 day culture. Based on our measurement and estimation of AuO exposure from temple workers, their maximum accumulation concentration of AuO is 289 nM in blood in 1 day, arguing that the environmental exposure of AuO could dramatically cause damage to the respiratory system.

Previous studies indicated that AuO causes DNA damage after in vitro treatment in culture cells and oral administration in rats and mice.^39^, ^40^ Different from previously experimental designs, low dose of AuO is used for mimicking an achievable exposure dosage in this study, consequently cell viability is not affected nor DNA damage is observed. Whether the damage effect is caused by direct AuO‐DNA interaction remains unclear. Recently, AuO is demonstrated to selectively bind the G‐quadruplex structure of DNA with higher affinity than single or double strand DNA.^41^ G‐quadruplex is a square planar structure formed in guanine rich nucleic acids such as telomeres through Hoogsteen hydrogen bond.^42^ Recent studies implicated that G‐quadruplex involves in the protection of telomere ends from nuclease attack,^43^ the programmed recombination of immunoglobin genes,^44^ and both positive and negative transcriptional regulation.^45^ This information suggests a potential mechanism that AuO induces ALDH1A1 expression through regulating the G‐quadruplex of ALDH1A1 gene. Using QGRS Mapper^46^ to predict the potential G‐quadruplex sequence, we indeed find G‐quadruplex sequences located at the promoter of ALDH1A1. However, the precise mechanism remains to be investigated.

Autophagy is an important mechanism used to clear pathogenic organism and deal with environmental stress such as starvation for maintaining cellular homeostasis. During the transformation of normal cells, autophagy protects genomic stability and inhibits the formation of chronic inflammatory microenvironment, thus preventing tumor generation.^47^ Autophagy has a complex and context‐dependent role in tumor cells, that is, autophagy suppresses primary tumor growth but is required for tumor maintenance and progression to advanced disease.^48^ Additionally, recent investigations have suggested that autophagy promotes multiple steps in the metastatic cascade.^30^ In this study, we find that AuO induces autophagy activity (Figure 3) as well as increasing the metastatic abilities of lung tumor cells to promote lung cancer malignancy, suggesting a positive correlation between autophagy and cancer metastasis, which is consistent with the current knowledge. Autophagy activity is not significantly induced in the bronchial epithelial cell line BEAS‐2B under low dose of AuO treatment (final 0.5 μM). Thus, we cannot evaluate whether the protective effect of autophagy involves in preventing AuO‐induced transformation or normal lung cell is intrinsically resistant to AuO. Does autophagy keep its protective ability or DNA damage cause malignant transformation in normal lung cell under heavy AuO exposure? These questions need to be further explored.

CSC defined as a subset of tumor cells with self‐renewability involves in cancer initiation and progression. CSC is also associated with the resistance to radiation and chemotherapy as well as the metastasis of carcinoma,^49^, ^50^ thus increased CSC population has been viewed as a poor prognosis indicator of cancer patients.^51^ ALDH1A1 overexpression has been known to associate with lung cancer malignancy.^29^ Our study reveals that ALDH1A1 plays an important role in mediating AuO‐induced stemness features and metastatic abilities of lung tumor cells, whereas ALDH1A1 knockdown significantly blocks these effects. In clinic, ALDH1A1 overexpression might be relevant to AuO exposure, especially in Asian countries with the custom of burning incense. Their correlation could be addressed clearly through epidemiological study in the future. In recent years, accumulating efforts have been put to develop drugs for targeting CSC.^52^ Since ALDH1A1 is crucial for AuO‐induced lung malignancy, ALDH1A1 targeting therapy could be benefit to the patients suffering from AuO exposure.^53^, ^54^

In conclusion, we identify AuO released from incense smoke as an unexpected air pollutant due to its thermostability. The damage of AuO to the respiratory system has never been awakened. In this study, we find that AuO promotes lung cancer malignancy through increasing autophagy activity and the stemness of lung cancer cells. AuO is currently used as an additive colorant in incense manufacture, to some degree meaning the control of its usage could be accessible. For public health, reducing or avoiding the use of AuO in incense is recommended.

## Supporting information

Additional Supporting Information may be found online in the supporting information tab for this article.

Supporting Information
