# Supplementary material for: Auramine O, an incense smoke ingredient, promotes lung cancer malignancy
Source: Environ Toxicol. 2017 Jul 19;32(11):2379–91. doi: 10.1002/tox.22451 (PMC5655719; doi:10.1002/tox.22451)
Supplement: Supplementary file 1 — Supporting Information [file TOX-32-2379-s001.pdf]

Suppl. Table 1

Supplemental Table **1**
The estimation of the maximum accumulation concentration of AuO in blood of a temple worker

(1) The respiratory volume in an 8 hr day:
The typical respiratory rate for a healthy adult at rest is 12-20 (average: 16) breaths per minute.
Each breath has a volume of about 0.5 L
So, in 8 hr, 8 x 60 x 16 x 0.5 = 3840 (L) = 3.84 m^3
(2) AuO inhalation of a temple worker in an 8 hr day:
According to our previous study (Lung and Kao 2003),
the average exposure concentration of PM_{10} in temples was around 600 $\mu g/m^3$,
approximately equal to 88 $\mu g/m^3$ of AuO (assuming AuO accounts for 14.7% of PM_{10}).
So, AuO inhalation in 8 hr, 3.84 x 88 = 338 (μg) = 1.27 μmol
(3) The maximum concentration of AuO in blood in one day:
Total blood volume in an adult (assuming 60 kg body weight),
60 x 1/13 =4.6 (kg) = 4.4 L
So, the maximum accumulation concentration in blood is:
1.27 μmol / 4.4 L = 289 nM
